# A study of primary health care service efficiency and its spatial correlation in China

**DOI:** 10.1186/s12913-023-09197-x

**Published:** 2023-03-13

**Authors:** Kangni Mei, Ruxin Kou, Yuqing Bi, Yuzhuo Liu, Jingwen Huang, Wei Li

**Affiliations:** 1 School of Public Health, Weifang Medical University, Weifang, 261053 Shandong China; 2 School of Management, Weifang Medical University, Weifang, 261053 Shandong China

**Keywords:** Primary health care institution, Service efficiency, Data envelopment analysis, Exploratory spatial data analysis, Spatial autocorrelation, Moran’s I

## Abstract

**Background:**

China’s primary health care system has undergone major changes since the new round of medical reform in 2009, but the current status of primary health care institution service efficiency is still unsatisfactory. The purpose of this study is to compare and evaluate the China’s primary health care institution service efficiency and provide a reference for improving the efficiency and promoting the development of primary health care institution.

**Methods:**

Based on panel data of 31 provinces (municipalities directly under the central government and autonomous regions) in mainland China from 2011 to 2020, using the super efficiency slack-based measure-data envelopment analysis model, to analyze the data from a static perspective, and the changes in the efficiency of primary health care services were analyzed from a dynamic perspective by using the Malmquist index method. Spatial autocorrelation analysis method was used to verify the spatial correlation of primary health care service efficiency among various regions.

**Results:**

The number of Primary health care institutions increased from 918,000 in 2011 to 970,000 in 2020. The average primary health care institution service efficiency in the northeastern region including Jilin (0.324), Heilongjiang (0.460), Liaoning (0.453) and northern regions such as Shaanxi (0.344) and Neimenggu (0.403) was at a low level, while the eastern coastal regions such as Guangdong (1.116), Zhejiang (1.211), Shanghai (1.402) have higher average service efficiency levels. The global Moran’s I showed the existence of spatial autocorrelation, and the local Moran’s I index suggested that the problem of uneven regional development was prominent, showing a contiguous regional distribution pattern. Among them, H–H (high-efficiency regions) were mainly concentrated in Jiangsu, Anhui and Shanghai, and L-L regions (low-efficiency regions) were mostly in northern and northeastern China.

**Conclusion:**

The service efficiency of primary health care institution in China showed a rising trend in general, but the overall average efficiency was still at a low level, and there were significant geographical differences, which showed a spatial distribution of “high in the east and low in the west, high in the south and low in the north”. The northwestern region, after receiving relevant support, has seen a rapid development of primary health care, and its efficiency was steadily improving and gradually reaching a high level. The average primary health care institution service efficiency in the northeastern region including the northern region of China was at a low level, while the average efficiency in the eastern coastal region and some economically developed regions was high, which also verifies the dependence and high symbiosis of primary health care institution service efficiency on regional economy.

## Background

Achieving Universal Health Coverage (UHC) is one of the goals of the World Health Organization (WHO) and has been included as a national development priority in many countries over the past decades. The health of the population depends on the health care system of the country, which was defined by WHO as all organizations, institutions, and resources whose primary purpose is to improve health [1], and Primary Health Care (PHC) is the key to meeting most of the health needs of a person throughout his or her life, and having a strong PHC system is what makes it possible to achieve universal health coverage. This feature of a PHC that entitles everyone to receive basic health care has led more and more countries to promote the implementation of improved PHC systems [2].

China as the largest developing country in the world and the country with the largest population, PHC institutions in China mainly composed of community health service centers (stations), street health centers, township health centers, village health offices, outpatient clinics, and clinics (infirmaries), which are not only responsible for the diagnosis and treatment of general diseases, but also for basic public health services.

China’s health care reform policy was introduced in 2009 and then achieved the goal of the reform. In 2009, China proposed a medical reform with the goal of achieving universal access to basic public health services, achieving universal coverage, providing residents with safe, effective, convenient and inexpensive medical services, equalizing basic public health services, strengthening the construction of PHC system.

The “Opinions on Deepening the Reform of the Medical and Health System” was issued, which proposed “establishing a mechanism of division of labor and collaboration between hospitals and community health services, guiding general medical treatment”, and also proposed to “establish a mechanism for the division of labor and collaboration between hospitals and PHC institutions, guide general medical treatment to the primary level, and gradually made PHC institution as the first option in medical treatment, graded the system of tiered diagnosis and treatment with two-way referral” [3].

With the development of the economy and convenient transportation, but also due to the long-term social inertia and medical habits of the residents, coupled with the government’s policy adjustment and implementation process is not inclusiveness, so that residents still focus on large medical institutions, resulting in the overload of large medical institutions, the lack of capacity and inefficient operation of PHC institutions. The World Health Report 2010 states that about 20–40% of health resources were wasted due to inefficiency, and that these wasted resources could be shifted to achieve universal coverage by improving efficiency, indicating that inefficient health care delivery systems were not fully utilizing resources and that governments could do more to improve efficiency and reduce health resource waste. Therefore, understanding the inefficiency of PHC institution is important for both government and public health policy makers.

In terms of efficiency measures, commonly used methods are non-parametric methods such as data envelopment analysis (DEA) and parametric methods such as stochastic frontier analysis (SFA). DEA is more widely used [4] and its main advantage is that it does not require assumptions in the form of functions and can include multiple inputs and outputs measured in different units at the same time. Since the mid-1980s, DEA has become a valid tool for measuring health care efficiency and has been widely used in PHC for the last two decades.

Since Sherman [5] first applied the DEA method to health care to evaluate the efficiency of health care services, many scholars have conducted studies related to efficiency evaluation using the more mature DEA method. Numbers of studies have analyzed the efficiency of health care in the Americas [6, 7], Western Europe [8, 9], and Asia [10, 11] to elucidate the efficiency of health care systems in different countries; Hollingsworth conducted a systematic review of efficiency measures in several aspects of health care [12]; Kocaman used [13] DEA methods to investigate; Ozcan [14] used dynamic network DEA model to analyze the efficiency of public health system and health care system in OECD countries; Mohamadi [15] used Malmquist index model to assess and analyze the performance of Iranian health system on health resource use performance of efficiency; Giménez [16] calculated the directional distance function and the global Malmquist-Luenberger index to analyze the efficiency of hospitals in the Colombian health system from both static and dynamic perspectives; When choosing a specific model, most scholars tend to use traditional DEA models such as the Banker, Charnes, and Cooper (BCC) model, and the Charnes, Cooper, and Rhodes (CCR) model to assess and analyze efficiency over a certain time period [17–19]. However, the accuracy of the efficiency values measured by traditional DEA models is relatively low, and exists a certain degree of underestimation and does not reflect the period trend of efficiency [20]. Some scholars have used multi-stage DEA models, slack-based measure (SBM) models and super-efficiency DEA models to evaluate and analyze efficiency [21–23]. Some scholars also choose dynamic models such as Dt-SBM model and Malmquist index for inter-period efficiency evaluation and analysis [24, 25].

The geographical size of China makes it impossible to ignore that geographical reasons may have some corresponding influence in PHC institution service efficiency, so this study introduced spatial analysis into study, to explore their mutual influence relationships in terms of spatial correlation.

## Methods

### Indicators

In terms of input–output indicator selection, most studies select the corresponding indicators from three types of health resources: human, financial, and material [18, 19]. In contrast, the service efficiency of PHC institutions is a complex system that requires consideration of many factors, which includes multiple input and multiple output indicators. The results of different index combinations may be significantly different [22, 23]. The inputs of PHC institutions are resources in several areas such as human, material, and financial resources, mainly including the number of employees, the number of health technicians, equipment and fixed assets, management costs and so on. The output indicators usually include the number of emergency visits, hospitalizations, surgeries, financial income, and overhead costs [26–31].

Based on the existing literature and the availability of the corresponding indicators, this study selected the number of PHC institutions (X1), the actual number of beds (X2) and the number of health technicians (X3) as input indicators, the number of consultations (Y1) and the number of discharges (Y2) as output indicators from two dimensions–-service volume and economic efficiency (Table 1).

**Table 1:** PHC institution service efficiency indicators system

Item	Specific Indicators	Explanation of Indicators	Significance of Indicators
Input	X1: Number of Health Institutions (unit)	PHC institutions in China mainly composed of community health service centers (stations), street health centers, township health centers, village health offices, outpatient clinics, and clinics (infirmaries)	Reflecting the input of medical and health material resources in each year in the study area
	X2: Number of Beds (unit)	The number of beds is the sum of the number of beds in various medical and health institutions each year	
	X3: Number of health technicians (person)	Health technicians mainly include registered nurses, licensed (assistant) physicians, pharmacists, health supervisors, technicians, and other health technicians	Reflecting the investment scale of medical and health human resources in each year in the study area
Output	Y1: Number of Outpatients and Emergency Visits (10,000 person)	Outpatient and emergency visits are the sum of outpatient visits and emergency visits	Reflecting the supply of medical and health services of the research subjects in that year
	Y2: Number of hospital discharges(10,000 person)	Refers to all discharges after hospitalization during the reporting period	

The input indicator data of this study from 2011–2020 were obtained from the statistical indicators of 31 provinces (municipalities directly under the central government and autonomous regions) in the China Health and Health Statistical Yearbook, because of inconsistent statistical standards, Hong Kong, Macao and Taiwan provinces were not included. The map data were based on public geographic data downloaded from the Institute of Geographical Sciences and Natural Resources, Chinese Academy of Sciences.

### Research methods

#### DEA

##### DEA- SE—SBM model

The DEA method is one of the more mature methods of nonparametric efficiency analysis methods [32, 33]. Its traditional models include CCR, BCC and other models, which have the following two drawbacks: firstly, they are all measured based on radial angles and do not consider the slack variables of input and output components, the number of discharges, bed utilization and other outputs do not vary proportionally in the PHC institution service efficiency which made the measurement results not accurately; secondly, the measured efficiency values range from 0 to 1; at the same time, the units that achieve efficiency (efficiency value of 1) cannot be compared with.

Tone K proposed a non-radial, non-angle slack-based measure (SBM) model based on the traditional DEA model, which can effectively compensate for the shortcomings of some traditional models that do not incorporate slack variables when measuring low efficiency and improve the accuracy [34]. Based on this, Tone K combined the SBM model with the super-efficiency model [33] and proposed the super-efficiency slack measurement model (SE-SBM), which can further compare decision units (DMU) that simultaneously reach an efficiency value of 1 to compensate for the second deficiency mentioned above.

Therefore, in this study, the DEA-SE-SBM model based on input volume measurement was used in order to accurately reflect the efficiency of primary health care institution service in 31 provinces in China. The formula is shown in (1).

1 $$\begin{array}{c}{\rho }^{*}=min\rho =\frac{1-\left({}^{1}\!\left/ \!{}_{m}\right.\right)\sum_{i=1}^{m}\frac{{s}_{i}^{-}}{{x}_{ik}}}{1+\left({}^{1}\!\left/ \!{}_{z}\right.\right)\sum_{r=1}^{z}\frac{{s}_{r}^{+}}{{y}_{rk}}}\\ s.t.\left\{\begin{array}{c}{x}_{ik}=\sum_{j=1,j\ne k}^{n}{x}_{ij}{\lambda }_{j}+{s}_{i}^{-}\\ {y}_{rk}=\sum_{j=1,j\ne k}^{n}{y}_{rj}{\lambda }_{j}-{s}_{r}^{+}\\ {\lambda }_{j},{s}_{i}^{-},{s}_{r}^{+}>0\\ i=\mathrm{1,2},\dots .,m\\ r=\mathrm{1,2},\dots .,z\\ j=\mathrm{1,2},\dots ,n;j\ne k\end{array}\right.\end{array}$$

*n* is the number of measured regions (*n* = 31 in this study), *j* is the *j*-th province (*j* = 1, 2, …, *n*), and each region has *m* inputs and *z* outputs. ${x}_{ij}$ and ${y}_{rj}$ denote the *i*-th input and *r*-th output of the *j*-th region. ${x}_{ik}$ and ${y}_{rk}$ denote the *i*-th input and *r*-th output of the *k*-th region. ρ* denotes the efficiency value of each region. *λ* denotes the weight vector; ${s}_{i}^{-}$ and ${s}_{i}^{+}$ are the slack variables of the input vector and output vector, which denote the savings of health care resource inputs and the increase of health care service outputs in the pilot regions, respectively. In addition, according to model (1), the service efficiency of PHC institution in 31 provinces in China from 2011—2020 can be calculated. As shown in Eq. (2), ${Score}_{kt}$ denotes the efficiency of health care services in city *k* in year *t*.

2 $${Score}_{kt}={\rho }_{kt}^{*}\left(k=\mathrm{1,2},3\dots ..31;t=\mathrm{2011,2012},\dots .,2020\right)$$

##### DEA-Malmquist model

In terms of efficiency evaluation, the DEA-SE-SBM model can only measure the efficiency within a certain time. It cannot continuously compare different time periods and cannot reflect the dynamic evolutionary characteristics of efficiency. Therefore, this study uses the DEA-Malmquist model to further calculate the total factor productivity change (TFPCH) of health services [35–37] to reveal its dynamic evolution.

Let (${x}^{t}$,${y}^{t}$,) and (${x}^{t+1}$, ${y}^{t+1}$) denote the input–output vectors of the health care system in periods t and t + 1, respectively, the TFPCH of the two adjacent periods are:

3 $$TFPCH=M\left({x}^{t},{y}^{t},{x}^{t+1},{y}^{t+1}\right)={\left[\frac{{D}^{t}\left({x}^{t+1},{y}^{t+1}\right)}{{D}^{t}\left({x}^{t},{y}^{t}\right)}\times \frac{{D}^{t+1}\left({x}^{t+1},{y}^{t+1}\right)}{{D}^{t+1}\left({x}^{t},{y}^{t}\right)}\right]}^\frac{1}{2}$$

${D}^{t}$(${x}^{t}$,${y}^{t}$) and ${D}^{t}$(${x}^{t+1}$, ${y}^{t+1}$) denote the distance function of the desired region in period *t* and period *t* + *1*, respectively, with period *t* serving as the technical reference. ${D}^{t+1}$(${x}^{t}$, ${y}^{t}$) and ${D}^{t+1}$(${x}^{t+1}$,${y}^{t+1}$) have similar meanings. According to the decomposition of the Malmquist index by Fare [38], it can be further decomposed into two components: the index of technical efficiency change (EFFCH) and the index of technical change (TECHCH). In addition, with variable returns to scale (VRS) as an assumption, EFFCH can be decomposed into pure efficiency change index (PECH) and scale efficiency change index (SECH). If TFPCH > 1, it means that the total factor productivity of the test subject has improved during t ~ t + 1. If TFPCH < 1, it indicates a decrease. If TFPCH = 1, it indicates that it remains unchanged. TECHCH > 1 represents technological progress and vice versa. PECH represents change in efficiency due to change in management level, and PECH > 1 represents improvement in management level and vice versa. SECH > 1 represents optimization of scale efficiency and vice versa.

#### Exploratory spatial data analysis

ESDA is a research method in spatial econometrics that is mainly used to study the correlation between a phenomenon and the attribute values of its neighboring units in geographic space [39], to measure the aggregation or dispersion of the spatial element attributes of the research object [40]. It mainly includes global spatial autocorrelation analysis and local spatial autocorrelation analysis [41]. In this study, global *Moran’s I* and local *Moran’s I* were used to reveal the overall spatial correlation and internal correlation characteristics of the service efficiency of PHC in China [42–44].

##### Global spatial autocorrelation

Global spatial autocorrelation analysis measures the degree of spatial correlation and variation among the study units from the overall level. GeoDa 1.18 software was used to calculate the global *Moran’s I* to quantify the overall spatial correlation of the service efficiency of PHC. The calculation formula is as follows:

4 $$I=\frac{n\sum_{j=1}^{n}\sum_{k=1}^{n}{w}_{jk}\left({\rho }_{j}^{*}-\overline{{\rho }^{*}}\right)\left({\rho }_{k}^{*}-\overline{{\rho }^{*}}\right)}{\sum_{j=1}^{n}\sum_{k=1}^{n}{W}_{jk}\sum_{j=1}^{n}{\left({\rho }_{j}^{*}-\overline{{\rho }^{*}}\right)}^{2}}$$

*I* is the global *Moran’s I*; ${\rho }_{j}^{*}$ and ${\rho }_{k}^{*}$ are the PHC institution service efficiency values of each region *j* and *k*. The ${W}_{jk}$ spatial weight matrix, which measures the spatial relationship between regions *j* and *k*; the Rook spatial adjacency method was used in this study. $\overline{{\rho }^{*}}$ is the average value of health service efficiency. The global *Moran’s I* take values in the range [-1,1][45]. When *I* > 0, it indicates that there is a spatial positive correlation in efficiency, the greater the spatial correlation; when *I* < 0, it indicates a spatial negative correlation, the more obvious the spatial difference. When *I* = 0, it indicates that there is no spatial correlation and exhibits a random distribution [46]. The *z*-value is also usually needed to assist in determining the significance of the Moran index.

##### Local spatial autocorrelation

Local spatial autocorrelation analysis is used to analyze the degree of spatial correlation between each spatial object and its neighboring units in a certain region to reflect the local characteristic differences in the distribution of spatial objects. It can be used as a supplement to the global spatial correlation analysis to compensate for the possible local spatial potential instability [47]. This study will reveal the degree of correlation between the PHC institution service efficiency in a province and its neighboring regions by calculating the local *Moran’s I*. For province *j*, the local *Moran’s I* index can be expressed as:

5 $${I}_{j}=\frac{n({\rho }_{j}^{*}-\overline{{\rho }^{*})}}{\sum_{j=1}^{n}{({\rho }_{j}^{*}-\overline{{\rho }^{*}})}^{2}}\sum_{k=1}^{n}{W}_{jk}\left({\rho }_{k}^{*}-\overline{{\rho }^{*}}\right)$$

The variables in the above equation have the same meaning as those in Eq. (4), when ${I}_{j}$ is positive, it means that the province is similar to its neighboring provinces in terms of attributes, and when ${I}_{j}$ is negative, it means that it is not similar. Meanwhile, four different spatial aggregation patterns were shown by plotting the Moran scatter plot and the Local Spatially Associated Aggregation Indicator (LISA) [48, 49].

The first and third quadrants in the Moran scatter plot represent spatial agglomeration effects, and the second and fourth quadrants represent spatial divergence effects. The first quadrant is the high agglomeration region (H–H agglomeration), which is used to represent the relationship of high efficiency provinces surrounded by high efficiency provinces; the third quadrant is the low agglomeration region (L-L agglomeration), which indicates that low efficiency provinces are surrounded by low efficiency provinces; the second quadrant (L–H agglomeration) is used to represent the relationship of low efficiency provinces surrounded by high efficiency provinces; and the fourth quadrant (H–L agglomeration) is used to represent the relationship of high efficiency provinces surrounded by low efficiency provinces The fourth quadrant (H–L aggregation) is used to represent the relationship of high-efficiency provinces surrounded by low-efficiency provinces.

### Data analysis

The study used Excel 2019 software to summarize the data; DEA- Malmquist index method was applied to the initial data by using DEAP 2.1 software to obtain the results of service efficiency for the years 2011–2020; MATLAB 2021B was used to conduct DEA-SE-SBM programming modeling and process the original data[50], to obtain the PHC service efficiency values of each region in the past ten years; The GeoDa 1.18.0 was used to establish the adjacency Rook matrix and bring in the service efficiency values of independent year regions to calculate the global Moran index and local Moran index to determine whether they have spatial autocorrelation. Finally, ArcGIS 10.8 software was used to visualize the data.

## Results

### Medical and health input–output in China

From 2011 to 2020, China’s overall investment in PHC increased significantly (Table 2). In terms of the number of health technicians, as China continues to strengthen the construction of health care personnel, the number of health technicians has increased significantly, with Guangdong and Jiangsu having the largest increases, from 210,938 and 192,143 in 2011 to 307,240 and 285,750 in 2020, respectively, an increase of 45.65% and 48.72%. From the point of view of the number of PHC institution, there are nine regions for negative growth, other regions for growth, including Shandong, the largest increase in the number of 15,172. In terms of the number of beds, Hainan had the largest increase (64.12%), followed by Guangxi and Jiangxi, up 63.87% and 63.30% respectively. In terms of the output of PHC institution service efficiency, Heilongjiang and Gansu saw the most significant decline in the number of visits, with growth rates of -42.17% and -23.88%, respectively. In terms of the number of hospital discharges, most regions showed a decline, with Tianjin showing a greater decline (-81.82%). Overall, most Chinese provinces have continued to increase their investment in PHC in the last decade, which is conducive to improving the accessibility of healthcare services. However, the output of PHC services in some provinces did not improve accordingly, especially in the number of hospital discharges, which showed a decreasing trend in most regions, and the efficiency of PHC resources utilization needs to be further studied.

**Table 2:** Comparison of input and output indicators of primary health care in 31 provinces (municipalities directly under the Central Government and autonomous regions) in China

Region	2011					2020				
	X1	X2	X3	Y1	Y2	X1	X2	X3	Y1	Y2
Beijing	8 718	4 423	54 468	5 259	4	9 675	5 145	86 400	6 919	1
Tianjin	3 981	6 883	21 690	3 253	11	5 258	6 007	36 689	4 043	2
Hebei	78 246	67 942	190 213	24 485	166	83 972	78 970	225 307	21 778	112
Shanxi	38 587	40 348	108 266	6 975	61	39 224	37 414	110 820	5 634	33
Neimenggu	21 905	23 992	66 747	5 364	45	23 277	26 769	78 094	4 311	24
Liaoning	33 712	34 236	97 272	8 110	66	32 174	38 346	103 145	6 891	34
Jilin	18 882	21 040	65 190	4 974	31	24 424	19 627	86 059	4 242	11
Heilongjiang	20 142	27 081	83 111	5 495	53	18 653	31 168	81 040	3 178	27
Shanghai	4 289	17 955	46 122	9 266	11	5 291	15 612	73 182	8 952	4
Jiangsu	29 659	68 097	192 143	23 055	169	32 703	101 630	285 750	28 069	222
Zhejiang	29 207	23 967	121 834	20 990	28	32 376	30 718	196 621	32 374	33
Anhui	21 434	56 291	133 737	13 525	143	27 400	79 745	169 973	21 689	117
Fujian	26 287	27 626	87 868	9 437	112	26 902	37 278	123 695	13 552	62
Jiangxi	38 063	38 211	111 183	12 539	218	35 214	62 398	122 870	13 322	169
Shandong	65 954	112 951	313 328	36 813	303	81 126	118 285	356 927	36 748	250
Henan	74 208	94 056	282 443	33 981	310	71 339	137 822	311 634	35 090	316
Hubei	34 509	62 330	154 855	17 444	192	33 852	98 093	178 432	16 292	265
Hunan	58 214	77 251	166 550	14 537	316	53 793	124 217	204 248	14 987	405
Guangdong	44 034	59 128	210 938	32 462	196	53 069	73 595	307 240	34 827	184
Guangxi	33 132	46 534	121 940	12 793	216	32 149	76 256	171 149	11 283	286
Hainan	4 513	5 831	18 626	2 241	11	5 733	9 570	31 009	3 079	6
Chongqing	17 037	37 196	77 760	8 264	145	19 838	55 901	102 150	8 995	199
Sichuan	73 646	113 909	228 917	26 802	436	79 491	149 620	285 911	29 333	458
Guizhou	24 957	34 256	73 043	7 663	185	27 138	54 113	124 823	8 459	130
Yunnan	21 800	40 488	84 633	11 178	121	24 592	61 932	154 144	14 868	162
Xizang	6 356	2 861	13 051	628	3	6 632	3 867	20 485	930	1
Shannxi	35 033	31 260	102 082	9 021	62	33 208	39 906	130 527	8 287	54
Gansu	25 884	25 154	67 831	7 680	59	24 597	32 896	74 988	5 846	72
Qinghai	5 608	4 325	15 610	1 172	16	6 018	5 398	20 138	1 072	9
Ningxia	3 886	2 781	11 660	1 458	7	4 247	4 094	19 915	1 793	4
Xinjiang	16 120	25 315	51 882	3 696	80	16 671	32 992	66 380	4 771	56

### Results of comprehensive evaluation of the efficiency of health care services

**Fig. 1:**
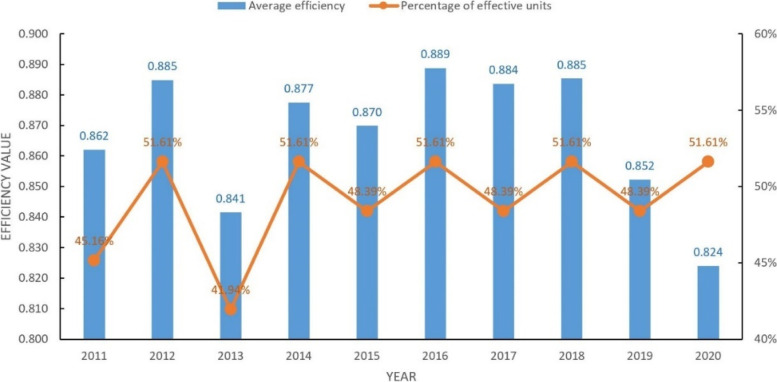
Average efficiency and effective unit percentage of service efficiency of primary health care institutions in China

Overall (Table 3), the average efficiency of PHC institution service in China from 2011—2020 is around 0.87. It indicates that there is still room to improve the overall efficiency of PHC institution service in China. Among them, the average service efficiency from 2014—2018 are relatively stable, but in 2019 and 2020 declined continuously (0.852 and 0.824). In terms of the annual effective unit share, the average service efficiency fluctuates from 2011—2013, and is more stable from 2014—2020, but is around 50% overall (Fig. 1), which means that the number of provinces with effective health services is around half of the number in the survey period of this study.

**Table 3:** Service efficiency values of primary health care institutions in China, 2011–2020

Region	2011	2012	2013	2014	2015	2016	2017	2018	2019	2020
Beijing	1.189	1.178	1.238	1.210	1.210	1.230	1.264	1.283	1.293	1.218
Tianjin	1.028	1.044	1.024	1.022	1.033	1.006	1.021	1.020	1.030	1.053
Hebei	0.650	0.640	0.612	0.620	0.660	0.689	0.639	0.618	0.537	0.499
Shanxi	0.352	0.343	0.322	0.349	0.355	0.371	0.385	0.369	0.322	0.266
Neimenggu	0.454	0.380	0.396	0.418	0.434	0.458	0.458	0.426	0.307	0.300
Liaoning	0.448	0.459	0.447	0.488	0.502	0.497	0.518	0.471	0.397	0.300
Jilin	0.381	0.385	0.294	0.318	0.304	0.316	0.389	0.370	0.284	0.193
Heilongjiang	0.468	0.507	0.527	0.500	0.494	0.528	0.500	0.414	0.371	0.287
Shanghai	1.453	1.416	1.404	1.408	1.413	1.451	1.417	1.413	1.352	1.294
Jiangsu	1.035	1.022	1.033	1.050	1.072	1.070	1.134	1.123	1.116	1.147
Zhejiang	1.167	1.156	1.150	1.178	1.220	1.226	1.231	1.242	1.243	1.302
Anhui	0.831	1.020	1.017	1.012	0.974	0.932	0.922	0.912	0.819	0.823
Fujian	0.821	0.802	0.720	0.711	0.706	0.702	0.685	0.701	0.703	0.626
Jiangxi	1.076	0.924	0.871	0.894	1.023	1.038	1.048	1.049	1.011	0.810
Shandong	1.066	1.092	1.101	1.066	1.040	1.019	1.012	1.025	0.745	1.025
Henan	1.032	0.906	0.840	0.941	1.006	1.006	1.014	1.002	0.881	1.031
Hubei	0.830	0.969	0.861	1.017	1.046	1.087	1.051	1.053	1.056	1.006
Hunan	0.816	0.776	0.802	1.028	1.056	1.056	1.025	1.093	1.059	1.082
Guangdong	1.176	1.175	1.194	1.209	1.185	1.187	1.147	1.141	1.115	1.051
Guangxi	0.957	1.009	1.126	1.101	1.071	1.045	0.923	0.922	1.034	1.043
Hainan	0.815	0.703	0.771	0.875	0.889	0.860	0.752	0.714	0.728	0.480
Chongqing	1.064	1.084	1.059	1.118	1.122	1.143	1.154	1.126	1.127	1.151
Sichuan	1.184	1.220	1.199	1.174	1.139	1.144	1.163	1.127	1.172	1.146
Guizhou	1.123	1.126	1.065	0.844	0.702	0.636	0.630	0.680	0.683	0.613
Yunnan	0.865	1.004	0.866	1.000	0.922	0.929	0.841	0.866	0.835	0.868
Xizang	0.372	1.032	0.354	0.376	0.367	0.450	1.002	1.016	1.027	1.020
Shannxi	0.460	0.511	0.488	0.537	0.549	0.557	0.590	0.589	0.527	0.407
Gansu	0.580	0.558	0.559	0.590	0.642	0.696	0.698	0.700	0.633	0.609
Qinghai	1.013	1.004	0.708	1.000	0.600	1.025	0.661	1.001	1.010	1.033
Ningxia	1.433	1.396	1.429	1.457	1.441	1.425	1.422	1.354	1.348	1.291
Xinjiang	0.585	0.590	0.607	0.691	0.787	0.777	0.695	0.626	0.655	0.570
Mean	0.862	0.885	0.841	0.877	0.870	0.889	0.884	0.885	0.852	0.824

**Fig. 2:**
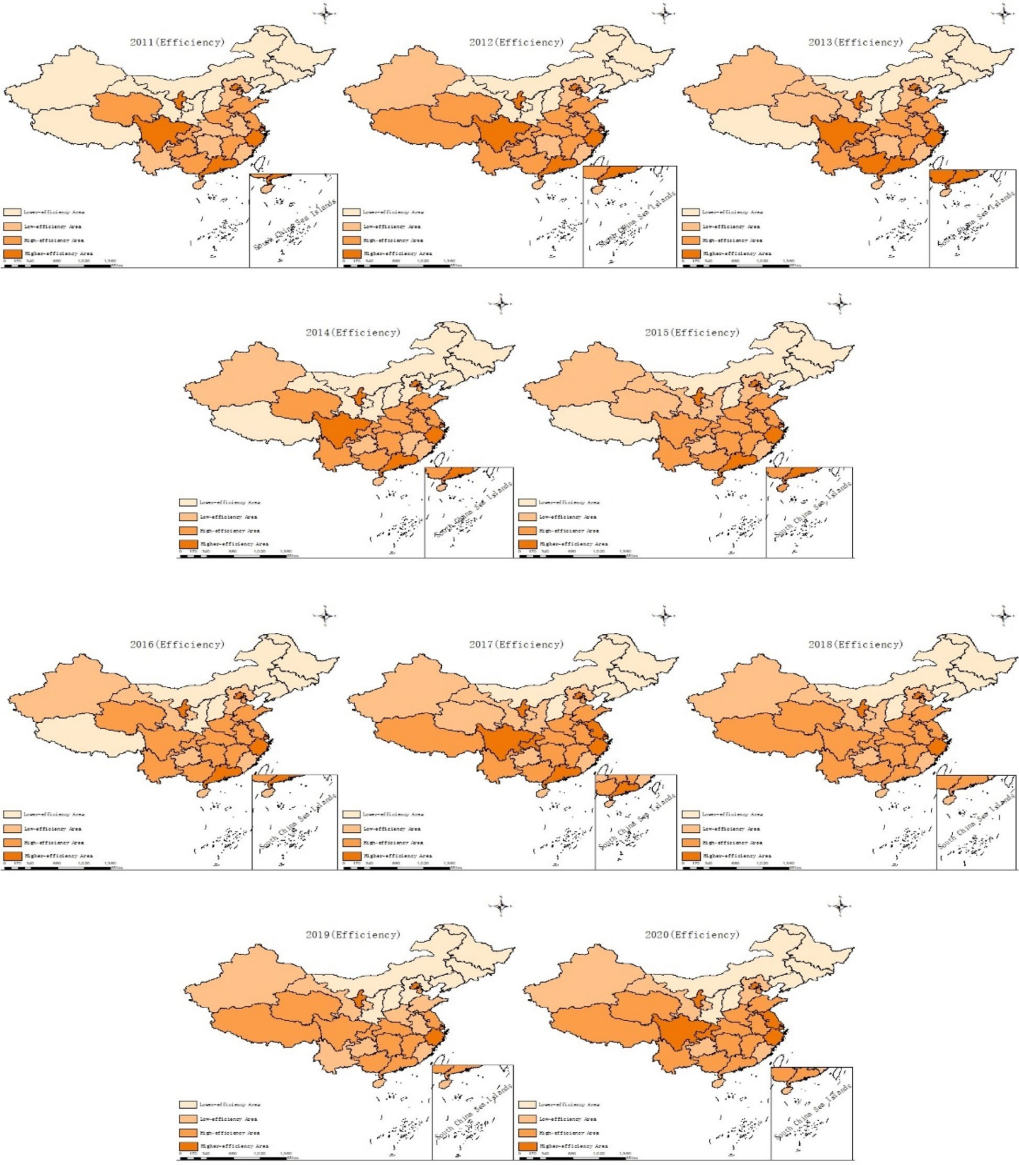
Distribution of efficiency levels of primary health care services in China (2011–2020)

In order to reflect the spatial distribution characteristics of the PHC institution service efficiency in China more clearly and intuitively, based on the efficiency values of each region in China from 2011 to 2020, ArcGIS 10.8 software was used to classify the 31 provinces in China into four categories: lower-efficient area, low-efficient area, high-efficient area, and higher-efficient areas (Fig. 2).

According to the efficiency distribution map for the decade 2011–2020, the pattern of efficiency distribution of primary health care services in China is relatively stable, and macroscopically it mainly shows a gradual enhancement from north to south. At the micro level, the regions with high service efficiency are still in the minority, and Beijing, Zhejiang, Sichuan, Gansu and Guangdong have maintained high service efficiency during the decade. Among the northwest regions, Xizang has made significant progress, gradually improving from the low efficiency level in 2011 and stabilizing at the high efficiency level, while Qinghai finally stabilized at the high efficiency level after alternating between the low efficiency level and the high efficiency level during the decade.

### DEA-Malmquist model

In terms of efficiency evaluation, this study uses the Malmquist index model to decompose the total factor productivity of the PHC institution service efficiency in China to further explore the dynamic evolution of efficiency and the deep-seated causes affecting the efficiency changes. Tables 4 and 5 showed the TFPCH and its decomposition in the time dimension and geographical dimension, respectively.

**Table 4:** Malmquist index and its decomposition for primary health care institutions in China (time dimension)

Year	EFFCH	TECHCH	PECH	SECH	TFPCH
2011–2012	1.020	1.037	1.007	1.013	1.058
2012–2013	0.978	1.033	0.996	0.982	1.011
2013–2014	1.012	0.962	1.014	0.998	0.974
2014–2015	1.004	0.961	1.000	1.004	0.965
2015–2016	1.019	0.988	1.006	1.012	1.006
2016–2017	0.994	1.003	0.99	1.004	0.997
2017–2018	0.959	0.978	0.988	0.97	0.937
2018–2019	0.951	1.003	0.97	0.98	0.953
2019–2020	0.97	0.872	0.986	0.984	0.845
Mean	0.989	0.981	0.995	0.994	0.970

**Table 5:** Malmquist index and its decomposition for primary health care institutions in China (geographical dimension)

Firm	EFFCH	TECHCH	PECH	SECH	TFPCH
Beijing	1	1.004	1	1	1.004
Tianjin	0.981	0.989	1	0.981	0.969
Hebei	0.981	0.981	0.977	1.004	0.963
Shanxi	0.983	0.978	0.997	0.986	0.961
Inner Mongolia	0.967	0.98	0.986	0.981	0.948
Liaoning	0.982	0.981	0.993	0.989	0.963
Jilin	0.954	0.984	0.975	0.978	0.939
Heilongjiang	0.954	0.971	0.973	0.981	0.927
Shanghai	1	0.973	1	1	0.973
Jiangsu	1.009	0.99	1	1.009	0.999
Zhejiang	1	1.009	1	1	1.009
Anhui	1.01	0.991	1.001	1.009	1.001
Fujian	0.99	0.961	0.993	0.997	0.952
Jiangxi	0.997	0.964	0.997	1	0.961
Shandong	0.996	0.984	1	0.996	0.98
Henan	1.009	0.983	1	1.009	0.991
Hubei	1.008	0.991	1.013	0.996	0.999
Hunan	1.026	0.97	1.005	1.021	0.995
Guangdong	0.992	0.987	1	0.992	0.98
Guangxi	1.005	0.979	1	1.005	0.984
Hainan	0.983	0.985	0.999	0.984	0.969
Chongqing	1	1.003	1	1	1.003
Sichuan	1.007	0.977	1	1.007	0.985
Guizhou	0.961	0.958	0.963	0.998	0.921
Yunnan	0.997	0.977	0.995	1.001	0.974
Xizang	0.976	0.984	1.003	0.973	0.961
Shanxi	0.979	0.983	0.984	0.995	0.962
Gansu	0.994	0.98	1.003	0.991	0.974
Qinghai	0.971	0.955	1	0.971	0.927
Ningxia	0.964	0.988	1	0.964	0.952
Xinjiang	0.995	0.963	0.995	1	0.958
Mean	0.989	0.981	0.995	0.994	0.970

As can be seen from Table 4, the average TFPCH of PHC institution service efficiency in China from 2011—2020 was less than 1 (0.986), and the total factor productivity declined at a rate of 3% per year, indicating that the overall efficiency of PHC institution service in China was not satisfactory, and the declining trend and characteristics have not improved significantly. Among them, TFPCH was the highest in 2011—2012 with 1.058, and total factor productivity increased by 5.8%. 2017—2018 and 2019—2020 had the most obvious decline in total factor productivity, which decreased by 6% and 10.8%, respectively.

From the decomposition index, the average values of EFFCH and TECHCH were less than 1 (0.989) and the results showed that both EFFCH and TECHCH have a declining trend, which jointly restrict the improvement of total factor productivity, and the impact of TECHCH on TFPCH was greater than that of EFFCH. The average values of PECH and SECH were 0.995 and 0.994 respectively, and PECH and SECH were declined at a rate of 0.5% and 0.6% per year, and the difference between the two was not significant. As can be seen from Table 5, the ten-year average total factor productivity of health services was increased in only four regions during the study period, including Zhejiang, Beijing, Chongqing and Anhui. Three of the provinces, Guizhou, Qinghai, and Heilongjiang, showed the largest decline of about -7%. Further analysis reveals that the main reasons hindering the growth of TFPCH in these provinces are the following: (1) The common constraint of declining EFFCH and TECHCH. There are 21 regions where both indices are < 1. (2) The decline of TECHCH. Shanghai, Jiangsu, Henan, Hubei, Sichuan and other regions TECHCH index decline, while EFFCH > 1. (3) decline in the index of technical efficiency change. For example, Jilin and Ningxia are mainly influenced by EFFCH, while the decline of TECHCH has a relative impact on them.

### Comprehensive evaluation results

**Fig. 3:**
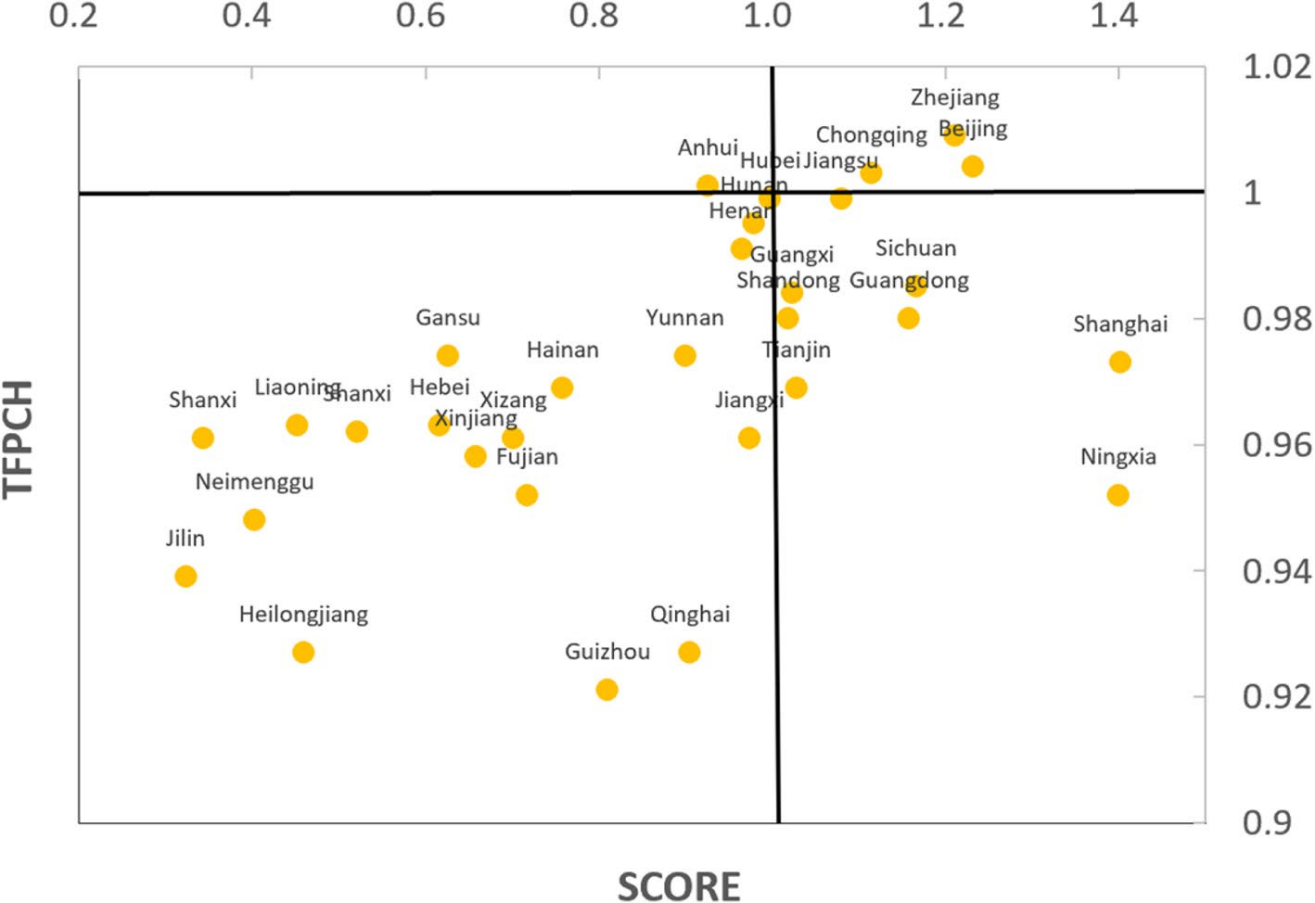
Scatter plot of efficiency values of DEA-SE-SBM model and total factor productivity index (TFPCH) of Malmquist index model

In order to fully reveal the static and dynamic distribution of the efficiency of PHC institution service efficiency in China in this study, the efficiency values (Score) based on the DEA-SE-SBM model and the total factor productivity index (TFPCH) based on the DEA-Malmquist index model for each region from 2011 to 2020 were plotted as quadrant distribution (Fig. 3).

The regions with an increasing trend of effective service efficiency were located in the first quadrant, including Zhejiang, Chongqing, Beijing and Jiangsu; the regions with an effective but decreasing trend of service efficiency were located in the fourth quadrant, mainly in Shanghai, Ningxia, Guangdong and Sichuan; the regions with an improving trend of health service efficiency, which have not yet reached the effective DEA status, were located in the second quadrant, only one region in Anhui. It is not difficult to find that there were more regions in the third quadrant (decreasing trend and ineffective service efficiency), accounting for 61.29% (19/31).

### Results of spatial correlation analysis of the efficiency of primary health care services

#### Results of global autocorrelation analysis

In this study, based on the efficiency values of PHC services calculated by the DEA-SE-SBM model, the global *Moran’s I* of the efficiency of primary health care services in 31 provinces in China was calculated using Geoda 1.18 software, and its *Z* statistical test value and *P* value were obtained to determine the overall spatial correlation of efficiency (Table 6).

**Table 6:** The global Moran’s I value of the efficiency of medical and health services in China from 2011 to 2020

Year	Moran’s I	Z	P
2011	0.262	2.4922	0.006
2012	0.336	3.1161	0.001
2013	0.312	2.9151	0.002
2014	0.291	2.7335	0.003
2015	0.321	2.9929	0.001
2016	0.275	2.5981	0.005
2017	0.290	2.7300	0.003
2018	0.347	3.2122	0.001
2019	0.384	3.5188	0.000
2020	0.344	3.1845	0.001

As can be seen from Table 6, the global *Moran’s I* for the service efficiency of PHC institution in each region of China was always positive during the study period, and all passed the significance test (*p* < 0.05), indicating that the services efficiency of PHC institution in each province of China showed a significant positive autocorrelation in space, which meant the provinces with similar efficiency of health care services across China were spatially aggregated or interdependent. The Moran index shows a volatile growth, and the spatial aggregation effect of overall health care service efficiency continues to increase.

### Results of local autocorrelation analysis

To further measure the specific location of agglomeration or dispersant, local spatial autocorrelation analysis was performed based on the global Moran’s I calculation. GeoDa 1.18 software was used to produce to obtain the Moran’s I scatter plot and ArcGIS software to produce the LISA agglomeration map to reflect the statistical characteristics and specific location of local spatial agglomeration of health service efficiency.

**Fig. 4:**
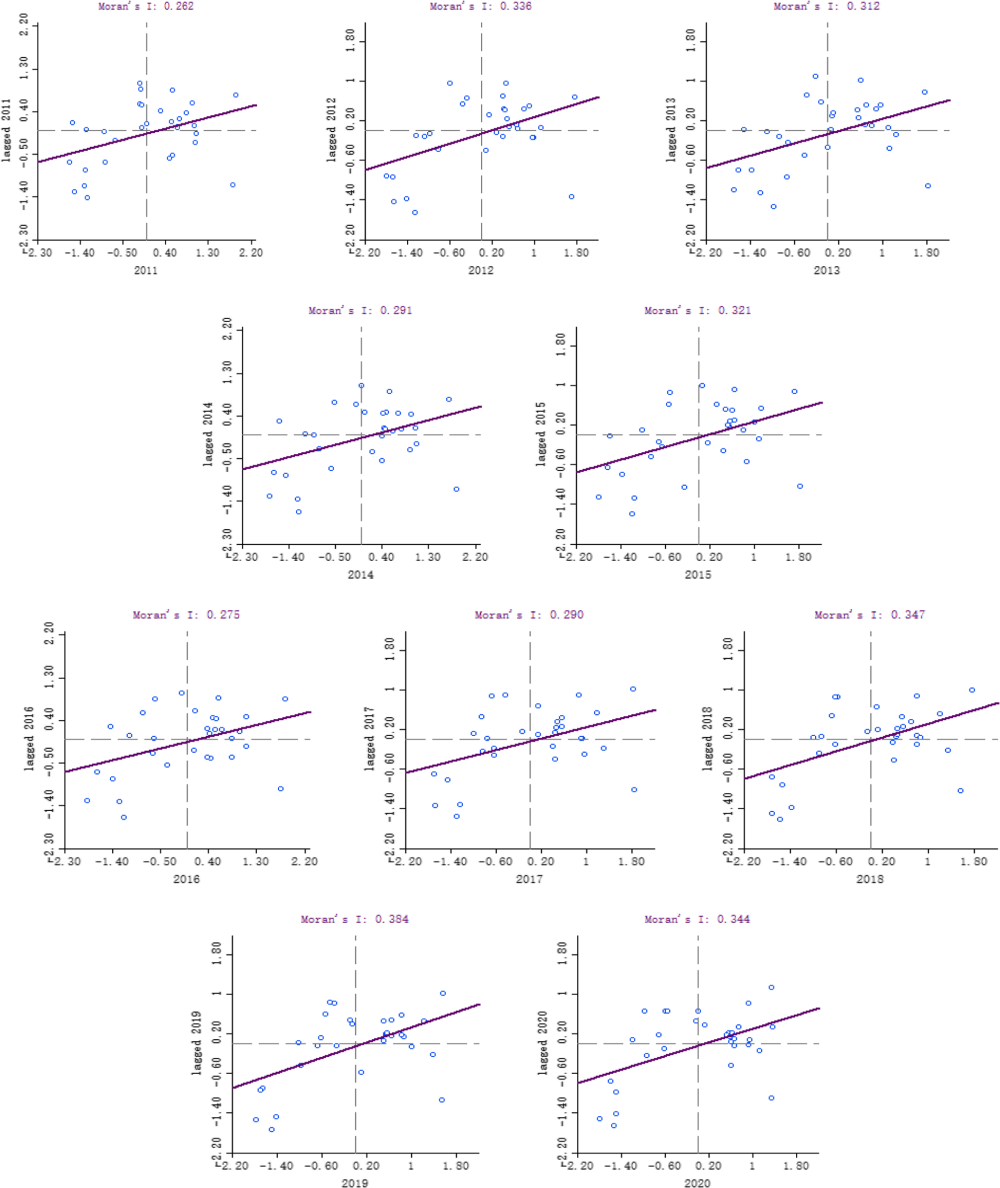
Moran’s I scatter plot

Combined with the Moran scatter plot (Fig. 4), it can be found that the services efficiency of China’s PHC showed a positive correlation with health care resource input. And most provinces were in the first quadrant and in the third quadrant (high aggregation area and low aggregation area) during the decade, and these provinces showed upward trend which further indicated that the spatial aggregation of the services efficiency of China’s PHC institution were good. In other words that provinces with high efficiency tended to converge with high-efficiency provinces, and low-efficiency ones tended to converge with efficient ones, and this phenomenon tended to increase. However, the number of L-L clusters was always more than H–H clusters, which indicated that the less efficient provinces in China had a higher degree of clustering than the more efficient ones, which has a negative impact on the overall efficiency.

**Fig. 5:**
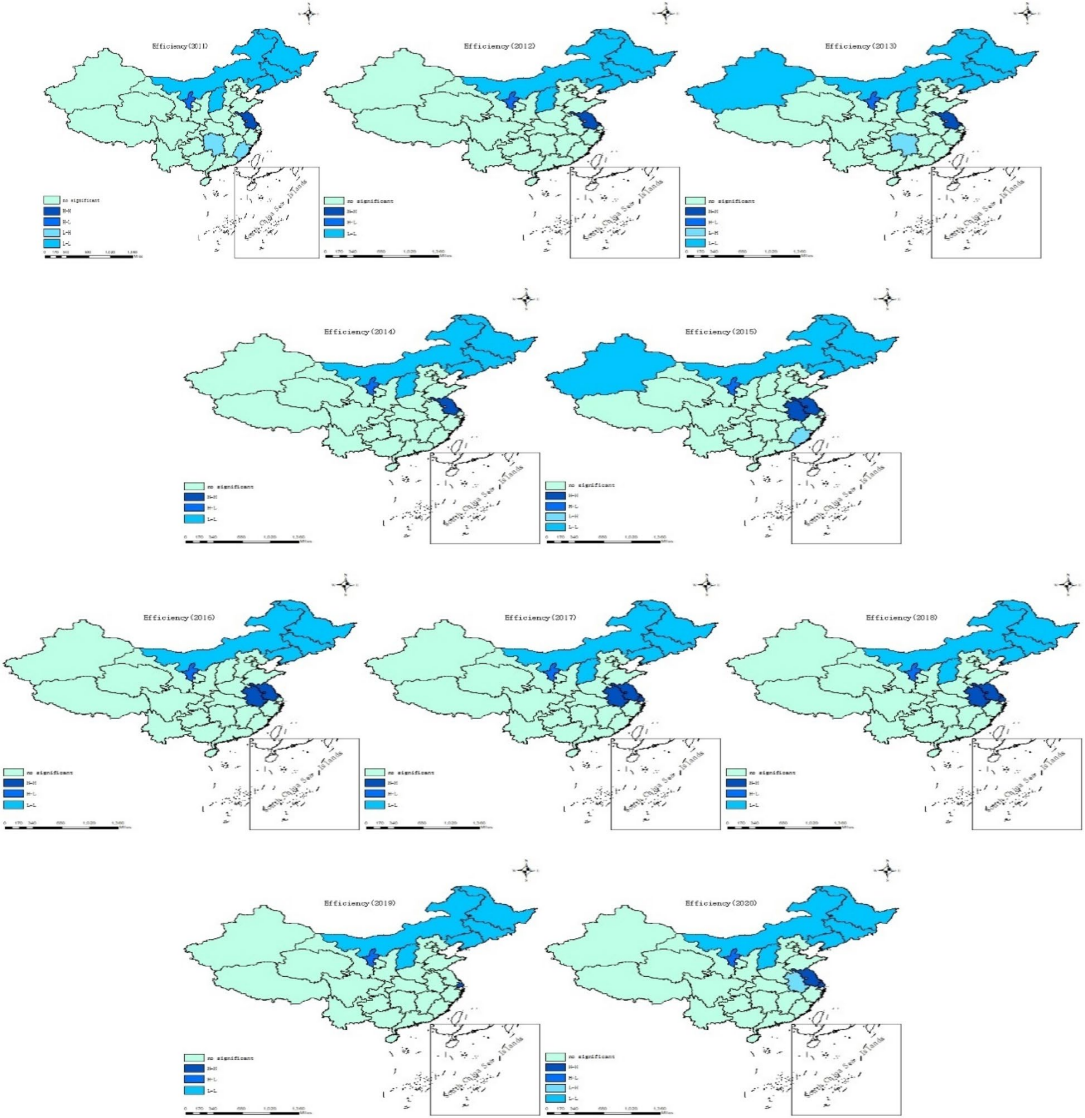
LISA aggregation map by location, 2011–2020

As can be seen from Fig. 5 and Table 7, the spatial aggregation characteristics of the provinces presenting significant levels were mainly of L-L type and there were distribution areas connected. Overall, the services efficiency of PHC in Neimenggu, Jilin, Liaoning, and Heilongjiang in the last ten years were L-L aggregation, and Shanxi was also L-L aggregation except in 2015 and 2016. Therefore, the above five provinces are divided into cold regions. In geographical view, these five provinces were all located in the northeast and northern China, indicating that the services efficiency of PHC institutions in those regions were much lower than that in other regions. The H–H gathering area mainly included the three regions of Zhejiang, Shanghai, and Jiangsu, so it can be classified as a hotspot area, indicating that the high-efficiency area of China’s PHC institutions services is mainly in the Yangtze River Delta region of China, which fits with the key economic development areas of China. And both in terms of geographical location and financial investment and economic development also confirmed the superiority of high-efficiency of PHC institutions’ services in this region. Ningxia was stable at the H–L level, indicating that the Ningxia is more efficient but had been surrounded by inefficient regions.

**Table 7:** 2011-2020phc H–H&L-L distribution regions

Year	H–H area	L-L area	L–H area	H–L area
2011	Jiangsu	Neimenggu, Shanxi, Jilin, Liaoning, Heilongjiang	Fujian, Hunan	Ningxia
2012	Jiangsu	Neimenggu, Shanxi, Jilin, Liaoning, Heilongjiang		Ningxia
2013	Jiangsu	Xinjiang, Neimenggu, Shanxi, Jilin, Liaoning, Heilongjiang	Hunan	Ningxia
2014	Jiangsu	Neimenggu, Shanxi, Jilin, Liaoning, Heilongjiang		Ningxia
2015	Jiangsu, Anhui	Xinjiang, Neimenggu, Jilin, Liaoning, Heilongjiang	Fujian	Ningxia
2016	Jiangsu, Anhui	Neimenggu, Jilin, Liaoning, Heilongjiang		Ningxia
2017	Jiangsu, Anhui, Shanghai	Neimenggu, Shanxi, Jilin, Liaoning, Heilongjiang		Ningxia
2018	Jiangsu, Anhui, Shanghai	Neimenggu, Shanxi, Jilin, Liaoning, Heilongjiang		Ningxia
2019	Shanghai	Neimenggu, Shanxi, Jilin, Liaoning, Heilongjiang		Ningxia
2020	Jiangsu, Shanghai	Neimenggu, Shanxi, Jilin, Liaoning, Heilongjiang	Anhui	Ningxia

## Discussion

The need to promote health system efficiency has been particularly emphasized in international and many national policies. Inefficiency and waste of health resources are among the major challenges facing health systems in China and other developing countries [51–53].

PHC institutions accounted for more than 90% of the number of health care institutions in China [54], and the China Health Statistics Yearbook showed that the number of PHC institutions had increased from 918,000 in 2011 to 970,000 in 2020, almost increase 5.67%. The financial revenue of PHC institutions increased from 263.84 billion yuan in 2011 to 751.97 billion yuan in 2020, with an annual growth rate of 12.34%. The service efficiency of China’s PHC institution generally showed an upward trend, but the service efficiency in local areas remained at a low level, and the overall average efficiency was low. After the new health care reform, the government invested a lot of money in the PHC system [55], which improved the accessibility of medical services, but residents’ choice of medical services still tended to be in upper-level hospitals [56, 57], and the situation of “upward concentration” of residents’ first choice in medical care did not change. Later, the government started to implement a hierarchical diagnosis and treatment system, and proposed a series of measures such as two-way referral services and reduction of the total cost of referral treatment. However, there had been little change in residents’ awareness of their preferred choice of health care, despite sufficient investment, the overall service utilization rate of PHC institution was low [58, 59], perhaps due to problems such as small development space of PHC institutions, low salary of employees and lack of adequate performance incentives. We suggest that PHCs should form a medical association with higher-level hospitals, provide promotion channels and training opportunities for PHC doctors, and benchmark their salaries and benefits with those of doctors of the same level in higher-level hospitals, to further promote the hierarchical treatment system and alleviate the phenomenon of “upward concentration” of medical treatment preferred.

The significant inequalities in geographical areas are indisputable. Like the findings of other researchers [60], capital resources are concentrated in the developed eastern provinces, where PHC resources including health technicians and health equipment are more abundant and very sufficient in terms of health inputs, and a large amount of input redundancy is underutilized. On the other hand, the western region has a limited level of technology, the economic development level in the west is also not as good as that in the east, the geographical area is larger, and the transportation is inconvenient, so people in the west are more inclined to go to PHC institutions. In addition, inefficiencies in the central region are reflected in the simultaneous existence of input redundancy and output deficiencies. Over time, the situation in the east has not changed [23], but the low service level of PHC institutions in the northwestern region has been well resolved, and after the policy tilt and increased investment in the southwest and northwest [61], their PHC institutions have been developed faster, and the development trend of PHC institutions has been very good during the decade, and their efficiency has been steadily improved, gradually reached a high efficiency level.

The global Moran’s I index of PHC service efficiency in 31 provinces in China from 2011 to 2020 were all positive, and they all passed the significance test and Z-statistic test, indicating that the efficiency of PHC services in each region of China is not randomly distributed in space, but has a certain spatial correlation. The efficiency of PHC services in China showed a spatial distribution of “high in the east and low in the west, high in the south and low in the north”. The local Moran’s I index indicated that the regional development imbalance was prominent. Combined with the LISA aggregation map, the spatial characteristics of PHC health service efficiency were mainly L-L and H–H aggregation at significant levels, and they were distributed in contiguous areas. The higher average service efficiency levels in the eastern coastal regions such as Guangdong (1.116), Zhejiang (1.211), Shanghai (1.402) and Chongqing (1.115) and Beijing (1.231) also verifies the dependence and high symbiosis of PHC services on regional economies [62], which were the early PHC reform pilot areas in China and the economic development areas in the Yangtze River Delta in China. The “ Matthew Effect “ of the medical system policies and public resources that have been formed in China in the early years has resulted in the gradual concentration of high-quality resources in economically developing regions, large cities, and large hospitals [63]. Therefore, the Health China 2030 [64] proposes to strengthen the support for the development of medical and health institutions in poor central and western regions. In this unbalanced interest pattern, when PHC institutions or large hospital groups in high-quality resource areas take the initiative to contact with areas with relatively backward resources or even completely backward areas (the health resource allocation in resource-developed areas flows to areas with relatively concentrated resources, and areas with relatively concentrated resources flow to areas with backward resources),this reverse resource flow method perhaps can break the existing “Matthew effect”.

The initiatives summarized in the reform pilot in Anhui, Jiangsu and Shanghai were vertical integration and upward -downward linkage. The formation of a win–win alliance or medical information sharing platform between higher level hospitals and PHC institutions, breaking through the medical barriers between PHC institutions and higher-level hospitals, reaching a helping alliance and synergistic win–win situation, solving the contradictions of unequal patient information and unfair distribution of health resources between higher and lower level hospitals, and realizing one-stop service for patients from diagnosis to treatment [65–67]. The pilot reform of China’s PHC institutions is gradually advancing from economically developed regions to undeveloped regions. Anhui, Jiangsu, and Shanghai as good demonstration cases can serve as teaching case templates for the rest of the provinces. The rest of the regions can explore and improve with the actual situation, and need the government to take the lead in introducing relevant policies, like improving the financial compensation, personnel establishment, remuneration and centralized procurement of drugs related to the vertical association of medical institutions. Those documents ensure the stability and sustainability of the vertical association.

## Conclusion

The study analyzed the service efficiency of China’s PHC institutions over a ten-year period from static and dynamic dimensions by using DEA- SE—SBM model and Malmquist index method, and based on this, spatial analysis was conducted to determine their geographical interactions and visualized them through cartographic soft. The study showed the positive trend of China’s PHC institution service efficiency during the decade, but the efficiency was generally at a low level. It also obviously showed regional differences. The efficiency of health services in PHC reform the pilot areas had been improved during the decade, which has alleviated the Matthew effect in public health to a certain extent, indicating that there is still more room for the development of China’s PHC institutions. Other inefficient regions can learn from the success stories of the initial reform pilot regions and propose reform programs to improve the efficiency of health services in their own regions, considering their own development characteristics.

### Insufficient articles

The study also has some limitations. The relevant data indicators are not very comprehensive and do not allow for the inclusion of other variations in efficiency impacts in the analysis. The efficiency results in this study reflected relative efficiency based on DEA calculations, using only the number of health resources and medical services. Secondly, different provinces, different populations (rural vs. urban), and different levels of information were not discussed in this study. Third, the study is limited to the impact on PHC service efficiency at the geographic level, leaving confounding factors such as socioeconomic performance, cultural differences, population movements, and differences in health insurance payment rates across provinces out of consideration.

## Data Availability

The data presented in this study are available on request from the corresponding author, and the data were not publicly available due to legal and privacy issues.

## Abbreviations

PHC: Primary health care
DEA: Data Envelopment Analysis
SFA: Stochastic Frontier Analysis
BCC: Banker, Charnes, and Cooper model
CCR: Charnes, Cooper, and Rhodes model
SBM: Slack-based measure model
ESDA: Exploratory Spatial Data Analysis
DMUs: Decision-making units
TFPCH: Total Factor Productivity Change index
EFFCH: Technical Efficiency Change index
TECHCH: Technical Change index
VRS: Variable returns to scale
PECH: Pure Efficiency Change index
SECH: Scale Efficiency Change index
LISA: Local Indicator of Spatial Association
